# Methylome Profiling of a Deuterostome Invertebrate Using Oxford Nanopore Technology (ONT)

**DOI:** 10.1111/1755-0998.70026

**Published:** 2025-08-05

**Authors:** Sarah Lok Ting Kwong, Alyssa Maree Budd, Julia Yun‐Hsuan Hung, Cecilia Villacorta‐Rath, Sven Uthicke

**Affiliations:** ^1^ Australian Institute of Marine Science PMB 3 Townsville MC Townsville Queensland Australia; ^2^ College of Science and Engineering James Cook University Bebegu Yumba Campus Townsville Queensland Australia; ^3^ Centre for Tropical Bioinformatics and Molecular Biology James Cook University Bebegu Yumba Campus Townsville Queensland Australia; ^4^ Environomics Future Science Platform, Indian Ocean Marine Research Centre Commonwealth Scientific and Industrial Research Organisation (CSIRO) Crawley WA Australia; ^5^ Centre for Tropical Water and Aquatic Ecosystem Research (TropWATER) James Cook University Bebegu Yumba Campus Townsville Queensland Australia

**Keywords:** *Acanthaster*, coral reef, DNA methylation, echinoderms, epigenetics, long‐read sequencing

## Abstract

DNA methylation is crucial for genome regulation and provides key insights into the interaction between genetics and environmental factors, offering valuable perspectives for ecological research. However, knowledge of DNA methylation patterns in nonmodel invertebrates remains limited. The present study addresses this knowledge gap by conducting the first methylome profiling of the Pacific crown‐of‐thorns seastar (CoTS; *Acanthaster* cf. *solaris*), a coral‐eating species that aggravates the decline of Indo‐Pacific coral reefs. Using Oxford Nanopore Technology (ONT) we generated long‐read sequences, covering over 90% of CpG dinucleotides in the CoTS genome. Our analysis revealed a mosaic methylation landscape with moderate genome‐wide methylation levels of 37.7%. Comparative analysis highlights the intermediate methylation state observed in other deuterostome invertebrates, positioning them between the hypomethylated genomes of protostomes and the hypermethylated genomes of vertebrates. Methylation in CoTS was predominantly localised within gene bodies, especially in intronic regions, enabling modulation of gene expression and potentially supporting fitness in dynamic marine environments. Additionally, elevated methylation in repetitive elements suggests a role in genome defence. This study demonstrates the effectiveness of ONT for comprehensive methylome analysis in ecologically important nonmodel species and deepens our understanding of the epigenetic landscape in deuterostome invertebrates. We also present a detailed laboratory and bioinformatics workflow, including modified phenol–chloroform protocols that address the challenge of extracting high molecular weight DNA from marine invertebrates. Together with the methylome profiles, these resources serve as a foundation for future research, enabling investigations into DNA methylation functions, applications for CoTS outbreak management and comparative studies across diverse lineages.

## 1 Introduction

DNA methylation, a key epigenetic mechanism, plays a pivotal role in regulating gene expression and chromatin structure (Buitrago et al. 2021). It involves the addition of methyl groups to DNA, typically at cytosine bases within cytosine–phosphate–guanine (CpG) sites, thereby modulating genomic activity without altering the underlying DNA sequence (Moore et al. 2013). In ecology, methylation studies are emerging as a valuable approach to reveal an additional layer of information about the complex interplay between genetic code and environmental factors, and how this interaction shapes phenotypic traits (Budd et al. 2022; Kilvitis et al. 2014; Verhoeven et al. 2016). For instance, DNA methylation plays a crucial role in stress responses and adaptation to environmental changes across various taxa from plants to marine invertebrates (Hawes, Fidler, et al. 2018; Kilvitis et al. 2017; Zhang et al. 2013). The exploration of DNA methylation has proven valuable, offering novel molecular insights and opening avenues for species management and conservation (Balard et al. 2024).

Despite advances in DNA methylation research, studies have predominantly targeted vertebrate species, leaving invertebrate methylation patterns comparatively understudied (de Mendoza et al. 2020). Vertebrates are widely recognised to follow a ‘global’ methylation pattern, with most CpG sites hypermethylated across the genome (Suzuki and Bird 2008), resulting in high average genome‐wide CpG methylation levels of 60% to 80% (Klughammer et al. 2023). In contrast, invertebrates exhibit a ‘mosaic’ methylation pattern, alternating between hypermethylated and unmethylated regions (Suzuki and Bird 2008). Their genome‐wide methylation levels vary across lineages, from nearly absent in *Drosophila melanogaster* and *Caenorhabditis elegans* (< 1%) (Rae and Steele 1979; Simpson et al. 1986) to moderate levels in more evolutionarily derived invertebrates, such as bivalves and cephalopods (10% to 40%) (Klughammer et al. 2023). This has led to the hypothesis that hypomethylation is the ancestral state of the epigenome (Tweedie et al. 1997), with the exception of the demosponge *Amphimedon queenslandica*, which exhibits vertebrate‐like hypermethylation, suggesting that this state may have evolved independently in some early metazoans (de Mendoza et al. 2019). However, the mechanisms, environmental drivers and evolutionary timeline of this process remain unclear (Suzuki and Bird 2008).

Many invertebrate species studied for their methylomes, such as the Western honeybee ( *Apis mellifera* ) and the Pacific oyster ( *Crassostrea gigas* ), belong to the Protostomia (Šrut 2021). In contrast, deuterostome invertebrates—including echinoderms, hemichordates, cephalochordates and tunicates—remain understudied despite being phylogenetically closer to vertebrates. Profiling the methylomes of deuterostome invertebrates therefore presents an opportunity to advance our understanding of DNA methylation across different lineages. Positioned phylogenetically between protostomes and vertebrates, deuterostome methylomes can shed light on the evolutionary trajectories of the DNA methylation landscape across metazoans. Furthermore, while previous studies on deuterostome invertebrates have often focused on selected genomic regions (Han et al. 2021; Strader et al. 2019), comprehensive methylome profiling provides significantly more information.

The functional roles of DNA methylation in invertebrates also remain poorly characterised. In vertebrates, DNA methylation is predominantly studied in CpG‐rich promoter regions, where hypermethylation typically leads to gene silencing (Al Adhami et al. 2022). The functional significance of DNA methylation in other genomic regions is less clear. Invertebrate methylation is primarily observed within gene bodies, where it is thought to play a crucial role in regulating splicing and preventing undesirable transcription from intragenic promoters (Sarda et al. 2012). Additionally, invertebrate DNA methylation is thought to contribute to genome defence by silencing transposable elements and other repetitive sequences, protecting the genome from potential instability and mutagenesis (Ying et al. 2022). Expanding research to include various invertebrates and thoroughly characterising their methylation profiles, including the identification of methylated genes and genomic regions, will enable a better understanding of the common principles and unique adaptations in the functional roles of DNA methylation across different lineages.

In recent years, Oxford Nanopore Technology (ONT) has emerged as a powerful tool for methylation analysis (Simpson et al. 2017). ONT works by measuring changes in electrical current as single DNA molecules pass through a nanopore, allowing direct detection of modified bases. This eliminates the need for bisulfite treatment, preserving DNA integrity and reducing errors from incomplete conversion (Sigurpalsdottir et al. 2024). In addition, the long‐read sequencing capability provides a transformative, contiguous view of both the genome and methylome (Jain et al. 2018), while the portable devices allow for real‐time, on‐site sequencing in diverse environments, benefiting field‐based ecological studies (Runtuwene et al. 2019). Although ONT initially faced high error rates, raw read accuracy now reaches 99.75% for super accurate (SUP) basecalling and 98.81% for cytosine methylation detection when using the recently released V14 chemistry (Lerminiaux et al. 2024; Oxford Nanopore Technologies 2023). Whole‐genome methylation profiling in nonmodel invertebrates remains rare, with data limited to selected representatives from Porifera, Cnidaria, Mollusca, Arthropoda and Echinodermata (Šrut 2021). These earlier efforts relied on whole‐genome bisulfite sequencing. To date, ONT has only been adopted in two cases—sea anemones (Dimond et al. 2021) and deep‐sea polychaetes (Perez et al. 2023) —both using pre‐V14 chemistry. By extending the application of these sequencing advancements beyond model organisms and biomedical research (González‐Recio et al. 2023; Sigurpalsdottir et al. 2024), we seek to demonstrate the feasibility of ONT as a practical tool for ecological methylation studies.

Our study focuses on the Pacific crown‐of‐thorns seastar (CoTS; *Acanthaster* cf. *solaris*), a species notorious for its extensive coral consumption during population outbreaks, posing a significant threat to Indo‐Pacific coral reefs (De'ath et al. 2012; Pisapia et al. 2016). Due to its impact, extensive research has been conducted on CoTS to develop local management strategies to control outbreaks and mitigate coral loss amid increasing climate change pressures (Pratchett et al. 2017). Despite these substantial efforts, numerous aspects of CoTS biology remain poorly understood, hindering effective management of population outbreaks (Pratchett et al. 2021). This indicates a pressing need for a more comprehensive understanding of the species, alongside new perspectives and innovative approaches. While the genome of CoTS has been annotated and is currently under investigation (Hall et al. 2017; Popovic et al. 2024), its methylome remains unexplored. Establishing a comprehensive profile of the CoTS methylome provides a foundation for understanding its epigenetic landscape. This can guide future research into critical epigenetic processes such as gene regulation, development and responses to environmental stress, potentially uncovering intervention points for controlling CoTS outbreaks.

**FIGURE 1:**
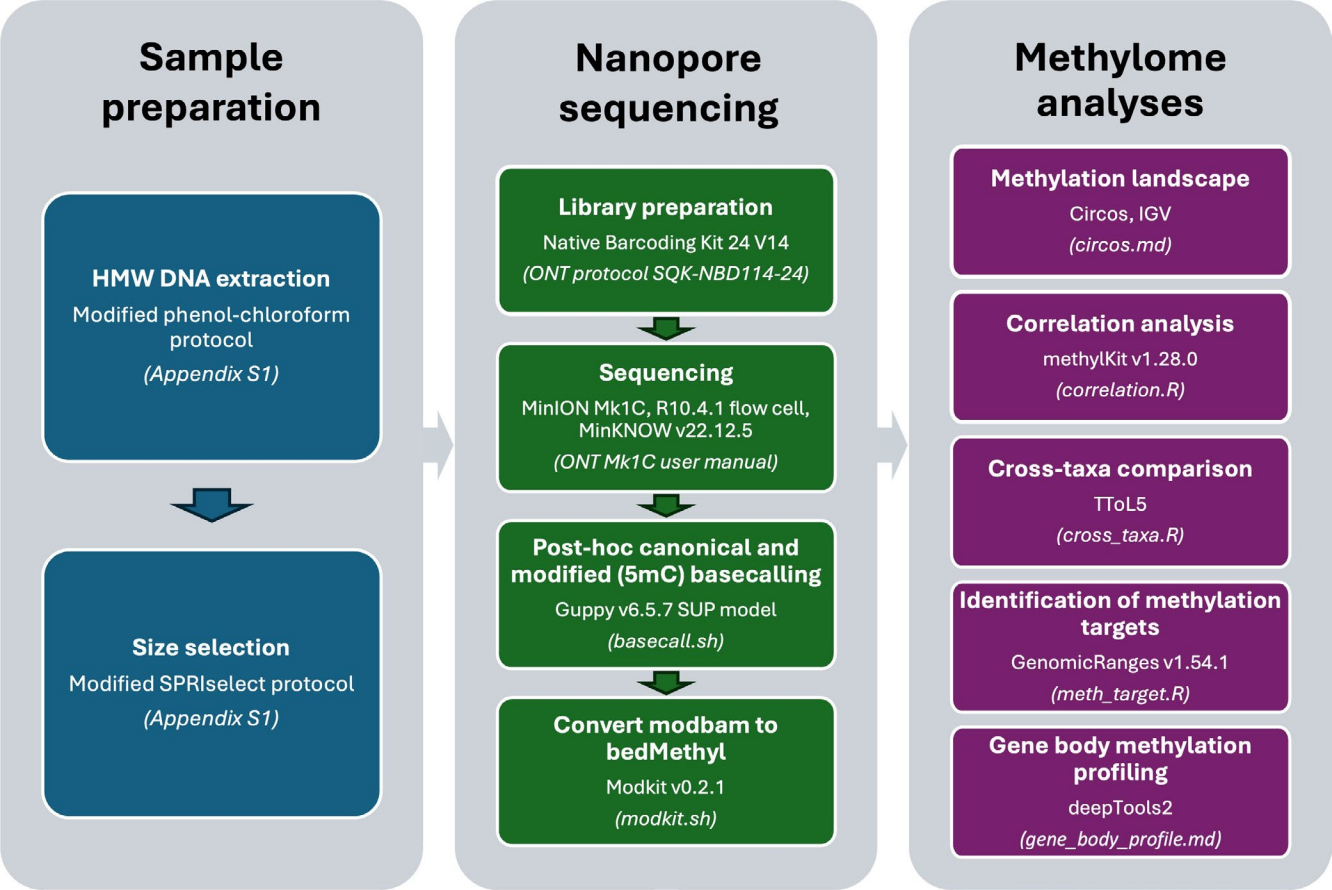
Schematic workflow for methylome profiling in the Pacific crown‐of‐thorns seastar (*Acanthaster* cf. *solaris*) using Oxford Nanopore sequencing. Key steps, materials and software tools are outlined at each stage. Laboratory protocols and bioinformatics scripts (in italics within brackets) are available in Appendix S1 and on GitHub (https://github.com/sarahkwong/cots_methylome/) with detailed workflow guidance. HMW, high molecular weight; SUP, super accurate.

The objective of this study is to leverage the ONT V14 chemistry advancements to profile the methylome of CoTS. We aim to demonstrate the utility of ONT for methylation detection in nonmodel species, supplementing the limited existing research in this area. The detailed workflow presented here, from high molecular weight DNA extraction to bioinformatics analysis, will support future methylation studies (Figure 1). In addition, we seek to establish a comprehensive methylome profile of CoTS, detailing methylation patterns and identifying methylation targets. By comparing the CoTS methylome with those of other taxa, we aim to better understand the evolutionary and functional implications of DNA methylation in deuterostome invertebrates.

## 2 Materials and Methods

### 2.1 Specimens

Field collected adult CoTS (*n* = 12; 6 females and 6 males) were spawned at the Australian Institute of Marine Science (AIMS) National Sea Simulator (SeaSim) in December 2020, using the method described in Uthicke et al. (2015). The seastars were settled and raised in aquaria, with an ontogenetic shift from crustose coralline algae to coral diet starting from 4 months postsettlement (Neil et al. 2022). In December 2022, when the samples were 2 years old, three biological replicates were randomly selected for sampling. We used laboratory‐reared individuals to ensure biological replicates were from the same cohort and of the same age, minimising between‐sample variability. Three replicates were used to reduce noise and distinguish biological signals from random variation, enhancing the reliability of the results. At the time of sampling, individuals had not yet entered the period of sex differentiation. Due to the absence of differentiated gonadal tissue, external sexual dimorphism, or known sex‐specific markers in CoTS, the sex of the specimens is unknown. Relatedness among the three specimens was assessed by genotype calling with ANGSD v0.940 using the following parameters: ‐minMapQ 20, ‐minQ 20, ‐doMajorMinor 1, ‐doMaf 1, ‐SNP_pval 1e‐4, ‐minMaf 0.05, ‐doHWE 1, ‐maxHetFreq 0.5 and ‐skipTriallelic 1. The output was then used to estimate kinship coefficients (φ) using the relatedness2 function in VCFTOOLS v0.1.16. Pyloric caeca tissue was collected from each specimen, snap‐frozen with liquid nitrogen and stored at‐80°C until DNA extraction.

### 2.2 High Molecular Weight DNA Extraction

Genomic DNA was extracted using a modified phenol–chloroform protocol (Sambrook and Russell 2006), incorporating increased proteinase K and EDTA concentrations, overnight lysis, and gentle handling to obtain high molecular weight DNA (Appendix S1), which was not achievable using commercial kits or the unmodified protocol. SPRIselect beads (Beckman Coulter) were used at 0.4× the sample volume to remove short DNA fragments (< 500 bp) prior to library preparation, to ensure optimal performance of Nanopore sequencing. The manufacturer's protocol was modified to ensure the complete removal of short DNA fragments (Appendix S1). Due to the limited yield of high molecular weight DNA (~150 ng per sample), extraction and size selection were performed on six samples per individual. DNA from the same individual was subsequently pooled for sequencing. The purity of the extracted DNA was assessed using the NanoDrop One Spectrophotometer (Thermo Fisher Scientific), and the DNA concentration was measured with the QuantiFluor dsDNA System (Promega). The integrity of the extracted DNA was visualised through 1% agarose gel electrophoresis.

### 2.3 Nanopore Sequencing

Nanopore sequencing libraries were prepared using the Native Barcoding Kit 24 V14 (SQK‐NBD114‐24) following the manufacturer's protocol. Sequencing was conducted on the MinION Mk1C platform implemented with MinKNOW v22.12.5, using the R10.4.1 flow cells (FLO‐MIN114). Each sequencing run spanned a duration of 72 h.

In this study, ‘methylation’ refers specifically to the modification of cytosine bases to 5‐methylcytosine (5mC), unless otherwise stated. Post hoc canonical and modified (5mC) basecalling was simultaneously conducted using the GPU version of Guppy v6.5.7 on a Linux computer with an NVIDIA A100 graphics card. The SUP model dna_r10.4.1_e8.2_260bps_modbases_5mc_cg_sup was applied with demultiplexing, barcode trimming, and alignment features enabled. This basecalling process resulted in modbam files, which are enhanced versions of BAM files that include the location and confidence score for base modifications. These modbam files contain reads with a mean Phred quality score above Q10, aligned to the CoTS OKI‐Apl_1.0 reference genome (Hall et al. 2017). Herein, any mention of the CoTS reference genome refers specifically to the OKI‐Apl_1.0 assembly (GCF_001949145.1). To summarise methylation status at each called CpG dinucleotide, the modbam files were converted to bedMethyl files using Modkit v0.2.1 with the ‘traditional’ preset. The pass confidence threshold value for the conversion was established at 99.4%, ensuring only reliable modification calls are considered. A minimum read depth of 5× was applied as a threshold for downstream analysis.

### 2.4 Methylome Analyses

Methylome analyses were performed using R version 4.3.2 (R Core Team 2022). The methylation level of individual CpG dinucleotides was defined as the ratio of methylated cytosines to the total number of reads covering that site. For broader genomic regions, the methylation level was determined by calculating the mean methylation across all CpG dinucleotides within the specified region. To visualise the overall methylation landscape, a Circos plot (Krzywinski et al. 2009) was generated to show the distribution of methylation levels, transposable elements, and gene density across the first 10 scaffolds of the CoTS reference genome (NW_019091355.1 to NW_019091364.1). These scaffolds were selected to highlight patterns without overcrowding the plot by including all scaffolds. However, note that the analysis was conducted across the entire genome and was not restricted to these selected scaffolds. To assess the correlation between specimens, we utilised the getCorrelation function from the methylKit package v1.28.0 (Akalin et al. 2012) to generate methylation distribution profiles, pairwise Pearson's correlation coefficients, and corresponding correlation plots. The Integrative Genomics Viewer (IGV) v2.16.2 (Thorvaldsdóttir et al. 2013) was used to visualise methylation patterns, with a representative snapshot captured from the beginning of the first scaffold of the CoTS reference genome (NW_019091355.1:1–200,000). To compare the CoTS methylome with other taxa, genome‐wide methylation levels were compiled and averaged from a diverse array of published studies including data from prebilaterian (cnidarian, poriferan), protostome (mollusc, annelid, arthropod), deuterostome invertebrate (tunicate, echinoderm, hemichordate) and vertebrate (mammal, bird, fish) species. The specific references for these data are listed in the legend of Figure 4 in the Results section. A phylogenetic tree was constructed using the fifth edition of the TimeTree of Life (TToL5), which synthesises divergence time estimates from multiple independent molecular clock analyses based on genetic data and fossil calibrations (Kumar et al. 2022).

To identify primary targets of methylation in the CoTS genome, various genomic regions were defined based on the National Center for Biotechnology Information (NCBI) RefSeq annotation of the CoTS reference genome (NCBI *Acanthaster planci* Annotation Release 100—August 7, 2017). Gene bodies were categorised into intronic and exonic regions, while promoters were defined as 2 kilobase pairs (kbp) upstream of the transcription start site (TSS). The remaining nonpromoter regions were classified as intergenic. Repetitive elements were identified using the soft‐masked regions of the CoTS reference genome. The GenomicRanges package v.1.54.1 (Lawrence et al. 2013) was employed to isolate these genomic regions to assess the distribution of CpG dinucleotides and their corresponding methylation levels. To assess whether the methylation levels differed between repetitive and nonrepetitive elements within intronic and intergenic regions, we performed nonparametric Wilcoxon rank sum tests. Methylation levels across gene bodies and their 2 kbp flanking regions were profiled using the plotProfile function in deepTools2 (Ramírez et al. 2016).

## 3 Results

### 3.1 Sequencing Performance

Three Nanopore sequencing runs were conducted, one for each of the three CoTS specimens. These runs generated a total of 22.32 gigabase pairs (Gbp) of data, encompassed by 9,634,060 reads (Table 1). This corresponds to an average coverage of 16× to 26× among specimens, given the size of the CoTS reference genome at 383.8 megabase pairs (Mbp). The maximum read length was 365,628 base pairs (bp). The read length N50 ranged from 3048 to 3572 bp, and the mean read length ranged from 2006 to 2694 bp. The mean Phred quality score across all reads was Q17.7, indicating an average accuracy of 98.3%.

**TABLE 1:** Summary of three Nanopore sequencing runs, each corresponding to an individual Pacific crown‐of‐thorns seastar (*Acanthaster* cf. *solaris*) specimen.

Specimen	Total reads	Total base pairs (bp)	CpGs covered (> 5×)	Max read length (bp)	Mean read length (bp)	Read length N50 (bp)
A	4,246,594	9,854,114,797	9,325,272	159,049	2320	3098
B	2,969,361	5,956,556,065	8,916,925	230,970	2006	3048
C	2,418,105	6,513,051,432	9,043,499	365,628	2694	3572

Reads from each specimen were aligned to the CoTS reference genome, resulting in mapping efficiencies from 83.8% to 85.0%. Of these aligned reads, 77.3% to 80.4% were uniquely mapped. Coverage analysis showed that 77.6% to 94.5% of the genome was covered at least 10×. The average mapping quality (MAPQ) score was 55.5 to 55.7, reflecting reliable alignment quality. The count of called CpG dinucleotides at read depth > 5× ranged from 8,916,925 to 9,325,272, representing coverage of 91.9% to 96.1% of the 9,705,479 CpG dinucleotides present in the CoTS reference genome.

### 3.2 Methylation Landscape

The relatedness analysis, based on 5725 SNPs, was interpreted according to the thresholds and ranges established by Manichaikul et al. (2010). It revealed a kinship coefficient (φ) of 0.15 between specimens 1 and 2, and 0.12 between Specimens 2 and 3, suggesting that they are likely half‐siblings (expected φ 0.088–0.177). The kinship coefficient between specimen 1 and 3 was 0.06, which falls within the range for third‐degree relatives (expected φ 0.044–0.088), and may have resulted from cryptic relatedness among broodstock.

**FIGURE 2:**
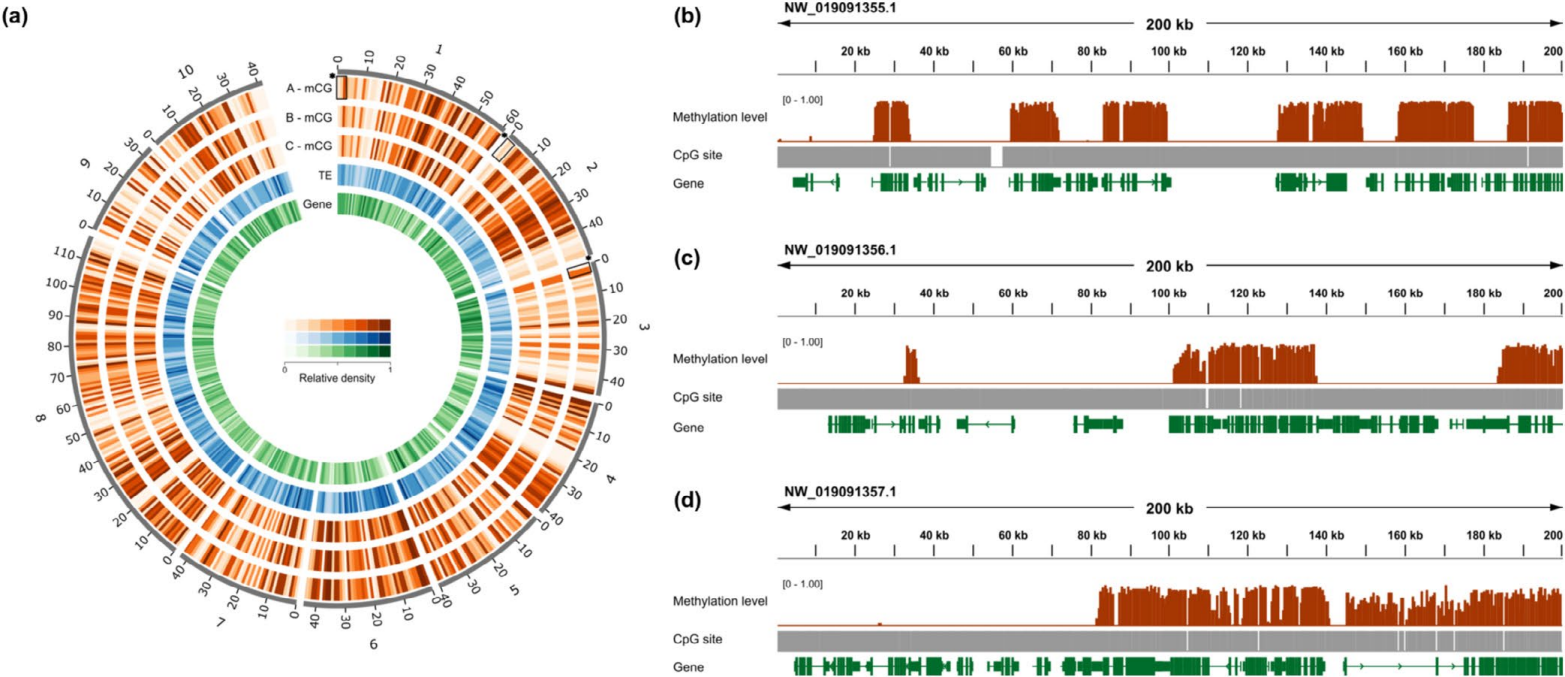
(a) Circos plot showing DNA methylation levels (mCG) in Pacific crown‐of‐thorns seastar (*Acanthaster* cf. *solaris*) specimens A, B, and C, along with transposable element (TE) density and gene density, across the first ten scaffolds of the CoTS OKI‐Apl_1.0 reference genome (NW_019091355.1 to NW_019091364.1). Tick marks around the circumference indicate genomic distances, with each unit representing 100 kb. (b–d) Zoomed‐in genome browser views of three distinct regions located on scaffolds 1, 2 and 3, respectively, corresponding to the areas indicated by the rectangular bracket and asterisk (*) in panel (a). These panels illustrate mosaic DNA methylation patterns observed across the genome. Tracks from top to bottom show: DNA methylation levels (range 0%–100%), represented by red bars; CpG sites positions, shown as grey vertical lines; and annotated genes, where green boxes represent exons and connecting lines indicate introns. Arrows denote the direction of transcription (5′ to 3′).

**FIGURE 3:**
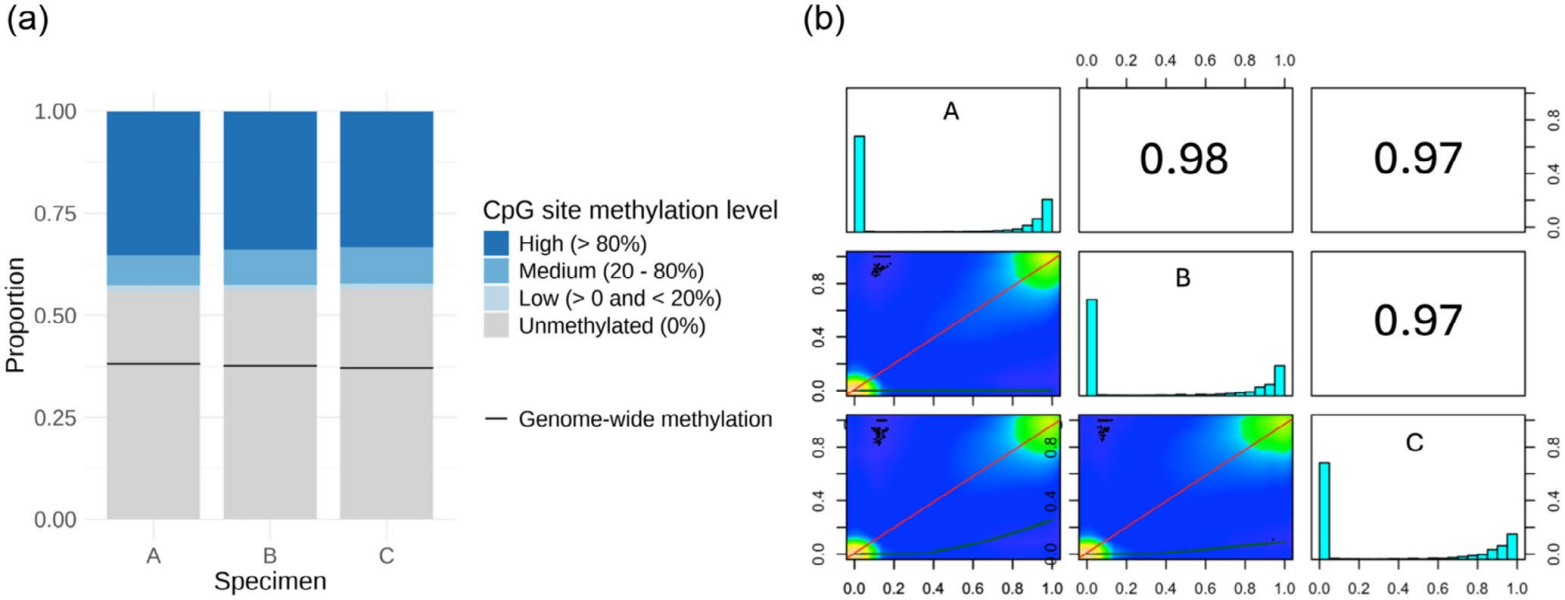
DNA methylation landscape of the Pacific crown‐of‐thorns seastar (*Acanthaster* cf. *solaris*). (a) Stacked bar plot showing the proportional distribution of CpG dinucleotide methylation levels and genome‐wide methylation across three specimens. (b) Pairwise comparison of methylation levels among specimens. Diagonal panels are histograms indicating the distribution of methylation levels for each specimen. Upper right panels display pairwise Pearson's correlation coefficients. Lower left panels are density plots of methylation levels between pairs of specimens, with warmer colours representing higher levels of similarity.

Examination of CoTS methylation profiles revealed a consistent pattern across specimens, with a moderately methylated genome and a patchy distribution of methylated regions. These features were visualised using a Circos plot (Figure 2a). In addition, mosaic methylation patterns were observed throughout the genome, characterised by alternating segments of heavily methylated and unmethylated regions (Figure 2b–d). In line with these observations, we found intermediate levels of genome‐wide methylation, ranging from 37.2% to 38.1% among the three individuals (Figure 3a). Over half (~56%) of the CpG dinucleotides in each specimen were completely unmethylated, with 34%, 8%, and 2% having high (> 80%), medium (20%–80%) and low (> 0 and < 20%) methylation levels, respectively (Figure 3a). Pairwise analysis showed that the methylation profiles were consistent among specimens, with Pearson's correlation coefficients ranging from *R* = 0.97 to 0.98 (*p* < 2.2e−16) (Figure 3b), indicating near‐identical patterns across individuals. Notably, this similarity was observed across all pairwise comparisons, with no apparent difference between those involving half‐siblings and those involving the third degree‐related specimens. These results also suggest a high degree of fidelity in methylation calling. The methylation distribution was bimodal, with most CpG sites exhibiting either no methylation or near 100% methylation (Figure 3b).

**FIGURE 4:**
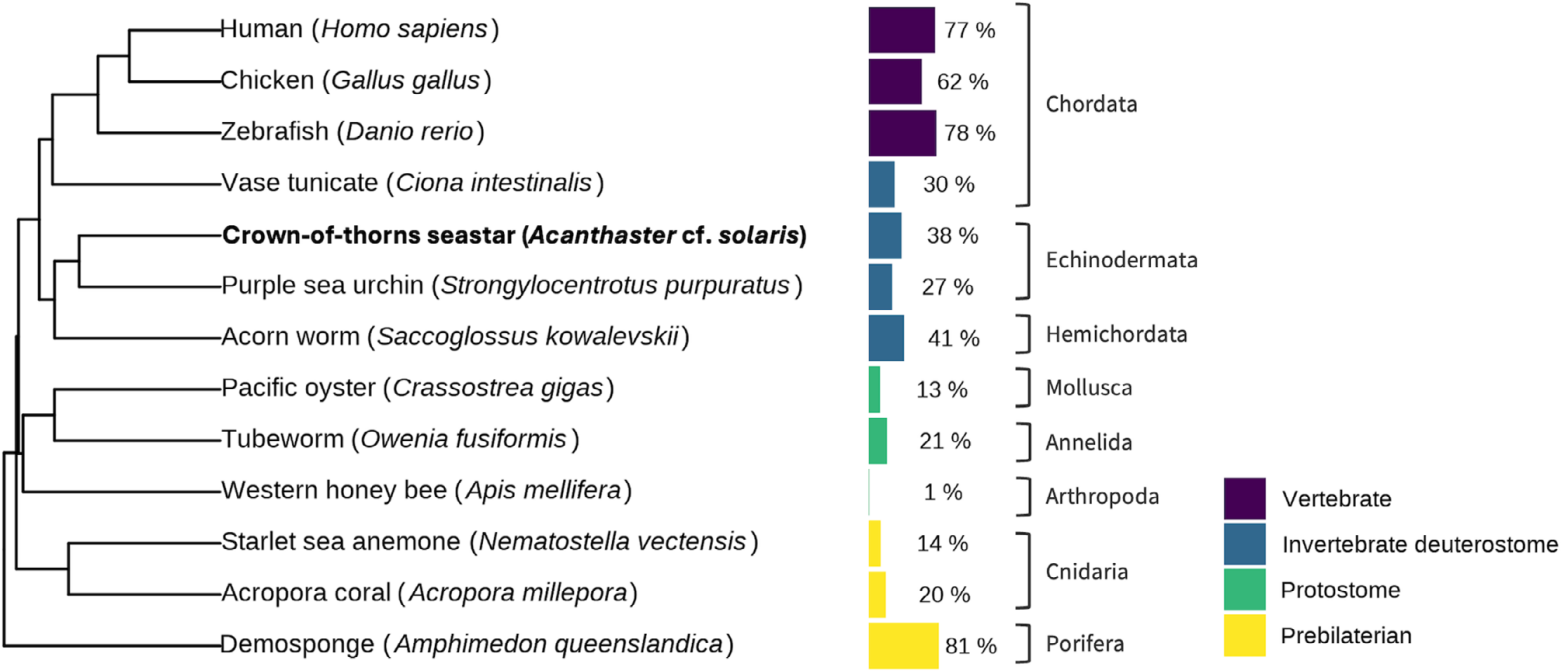
Phylogenetic tree comparing average genome‐wide DNA methylation across a diverse array of taxa, with methylation levels for each species indicated by bar plots. References: *Homo sapiens* (Schultz et al. 2015); *Gallus gallus* (Zhang et al. 2017); *Danio rerio* and *Amphimedon queenslandica* (de Mendoza et al. 2021); *Ciona intestinalis* , *Saccoglossus kowalevskii* and *Crassostrea gigas* (Klughammer et al. 2023); *Strongylocentrotus purpuratus* (Skvortsova et al. 2022); *Owenia fusiformis* (Guynes et al. 2024); *Apis mellifera* (Xu et al. 2019); *Nematostella vectensis* and *Acropora millepora* (Ying et al. 2022).

The genome‐wide methylation level in CoTS, compared to a diverse array of taxa, was visualised using a phylogenetic tree (Figure 4). The methylation level in CoTS was within the range of approximately 30% to 40%, aligning with other deuterostome invertebrates (vase tunicate, purple sea urchin, acorn worm; Figure 4). This level was higher than that observed in protostome invertebrates (Pacific oyster, tubeworm, western honeybee), which exhibited a broader range of methylation levels, from 1% in arthropods to 21% in annelids. In comparison with prebilaterians, CoTS showed higher methylation levels than Cnidarians (14% in the starlet sea anemone and 20% in Acropora coral), but demosponges stood out as a notable exception to all other groups with a distinctively high methylation level (81%). Vertebrates demonstrated the highest methylation levels across the taxa, reaching nearly 80%. This phylogenetic analysis positioned CoTS and deuterostome invertebrates as intermediates in the evolutionary spectrum of DNA methylation.

### 3.3 Primary Targets of Methylation

**FIGURE 5:**
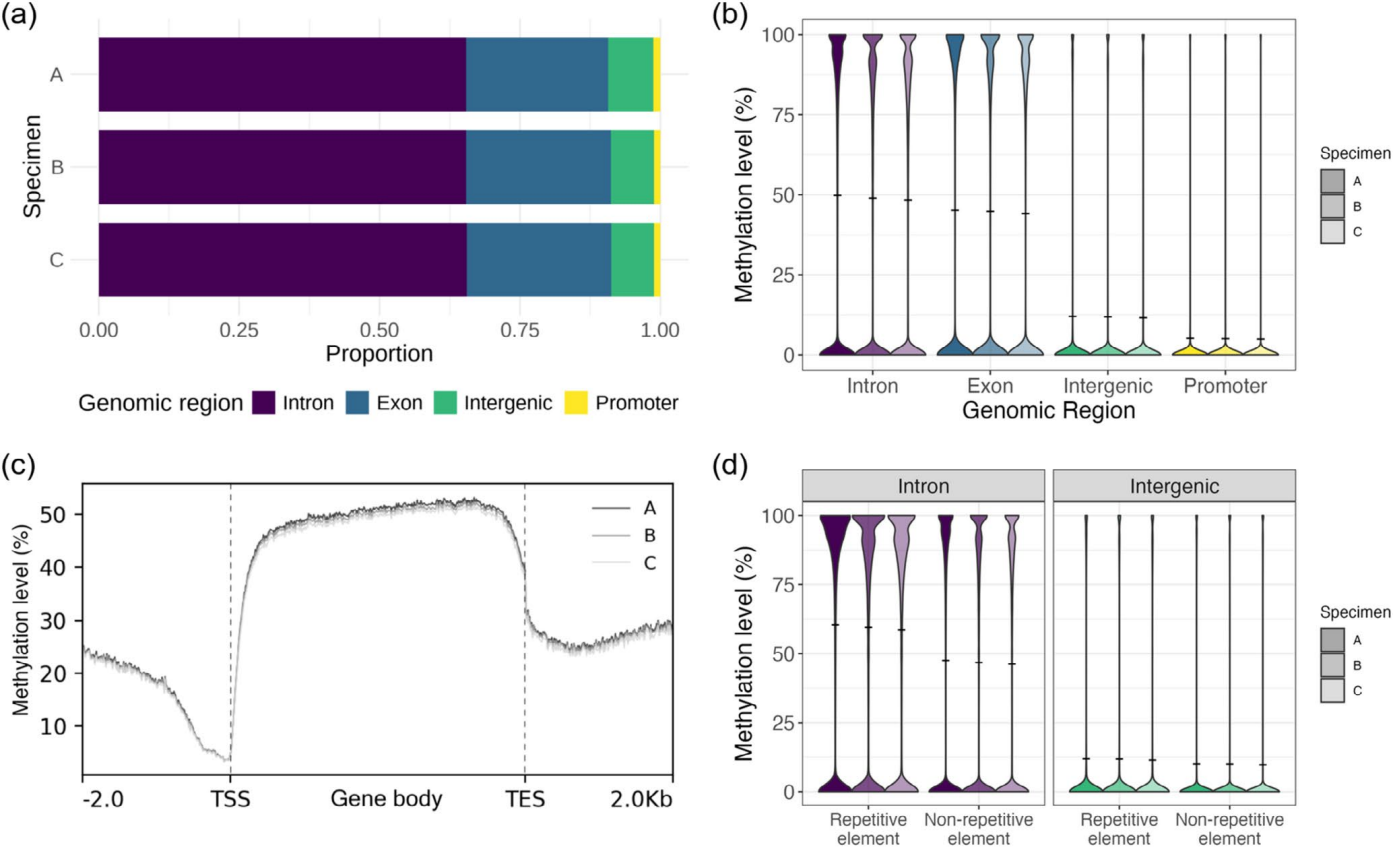
DNA methylation targets in the Pacific crown‐of‐thorns seastar (*Acanthaster* cf. *solaris*) genome. (a) Stacked bar plot illustrating the distribution of methylated CpG dinucleotides across genomic regions for each specimen. (b) Violin plot illustrating the distribution of methylation levels across genomic regions. Horizontal lines indicate mean methylation levels. (c) Average methylation profiles across gene bodies and 2 kb upstream and downstream flanking regions. Gene body lengths were normalised and aligned at the transcription start site (TSS) and transcription end site (TES). Methylation levels were averaged across all genes. (d) Violin plot illustrating the distribution of methylation levels in repetitive and nonrepetitive elements, within intron and intergenic regions. Horizontal lines indicate mean methylation levels.

We assessed the methylation status across various genomic regions to identify key methylation targets within the CoTS genome. Our analysis revealed that most methylated sites were located within gene bodies: 65% in intronic regions and 26% in exonic regions (Figure 5a). Only a small fraction of methylated dinucleotides was found within intergenic (8%) and promoter regions (1%). Genic regions exhibited relatively high methylation levels, with introns showing the highest level (48.4% to 49.8%), followed by exons (44.1% to 45.2%) (Figure 5b). These levels were four to tenfold higher than those observed in nongenic regions, where intergenic regions exhibited methylation levels of 11.5% to 12%, and promoter regions had the lowest level at 4.9% to 5.1% (Figure 5b). The top 50 most highly methylated genes, along with their gene descriptions and functions, are provided in Table S1. These genes are predominantly associated with fundamental cellular processes such as signal transduction, cellular structure and adhesion.

Profiling of genes and their flanking regions further revealed that methylation levels were higher within gene bodies compared to region 2 kbp up and downstream (Figure 5c). Methylation levels sharply increased following the TSS, remained elevated throughout the gene body, and then declined towards the TES. Additionally, we found that repetitive elements consistently exhibited higher methylation compared with the nonrepetitive background methylation in both intronic (*W* = 1.24 × 10^13^, *p* < 2.2 × 10^−16^) and intergenic regions (*W* = 4.22 × 10^12^, *p* < 2.2 × 10^−16^), as determined by Wilcoxon rank sum tests (Figure 5d). In intronic regions, the background methylation level averaged 46.8%, whereas repetitive elements showed higher levels at 59.5%. In intergenic regions, the overall methylation level was lower, with background methylation averaging 10% and repetitive elements showing a slight elevated level of 11.8% (Figure 5d).

## 4 Discussion

The present study provides the first comprehensive methylome profile of CoTS, a coral‐eating deuterostome invertebrate that plays a significant role in the decline of Indo‐Pacific coral reefs. Using ONT, we generated high‐quality long‐read sequences that capture over 90% of CpG dinucleotides within the CoTS genome. Our analysis revealed a mosaic methylation landscape characterised by a moderate genome‐wide methylation level of 37.7%, with gene bodies and repetitive elements being the primary targets of methylation.

Here, we demonstrate the utility of ONT as a robust method for comprehensive genomic and methylation analysis in nonmodel species. By covering over 90% of all CpG dinucleotides in the CoTS reference genome (Hall et al. 2017), we provide a genome‐wide perspective on the methylation profile, surpassing traditional methods such as Reduced Representation Bisulfite Sequencing (RRBS) and microarray, which capture less than 10% of all CpG sites and are biased towards GC‐rich regions (Sun et al. 2015). While whole‐genome bisulfite sequencing also offers genome‐wide coverage, ONT presents distinct advantages, such as preserving DNA integrity by avoiding bisulfite treatment, the ability to extract both genetic and epigenetic information, and allowing direct detection of different DNA modification types. There has been limited exploration of ONT for methylation studies in nonmodel invertebrates, with very few examples such as deep‐sea polychaetes (Perez et al. 2023) and sea anemones (Dimond et al. 2021). Our research not only contributes to the growing body of work validating ONT for ecological methylation studies, but also marks significant progress in efficiently generating reliable methylation data, facilitated by the use of the latest tools with improved accuracy. While our aim was to sequence the CoTS methylome, we also generated long‐read genomic data averaging over 2 kbp. This is made possible by optimising the extraction and size selection protocol to overcome the challenges of obtaining high molecular weight DNA from marine invertebrates (Panova et al. 2016). These long reads will facilitate future efforts to improve genome assembly and analysis of complex regions such as repeats, structural variants and GC‐rich regions that are challenging to resolve using short read sequences. By applying the protocols established here, we anticipate that future studies will continue to profile high‐quality methylomes across various taxa, advancing our understanding of methylation patterns and their functions in natural populations.

The CoTS methylome landscape observed in this study provides empirical evidence supporting the intermediate position of deuterostome invertebrates, situated between the hypomethylated genomes of most protostome invertebrates and the hypermethylated genomes of vertebrates. This evidence aligns with previous observations using CpG density as a proxy for methylation (Okamura et al. 2010) and enhances our understanding of the evolutionary dynamics of DNA methylation. While vertebrate‐like hypermethylation has been demonstrated in a species of demosponge (de Mendoza et al. 2019), this landscape has been lost in most prebilaterians and protostome invertebrates, which display mosaic methylation primarily in gene bodies and repetitive elements interspersed among hypomethylated regions (Feng et al. 2010). Our data, combined with the limited deuterostome methylation data available (Feng et al. 2010; Klughammer et al. 2023; Zemach et al. 2010), suggest an evolutionary reacquisition of methylation complexity in deuterostome invertebrates. This intermediate state may represent a unique balance between the flexibility of mosaic methylation and the regulatory robustness of hypermethylation. Overall, our observations are consistent with the hypothesis that methylation patterns have evolved modularly, with diverse strategies employed by different lineages to adapt their methylation landscapes to specific evolutionary pressures (Suzuki and Bird 2008).

Analysis of CoTS methylation profiles performed here revealed a moderate and mosaic pattern. While DNA methylation profiles are largely established early in development and follow predictable, species‐ or lineage‐specific patterns, certain regions may exhibit plasticity under specific environmental or physiological conditions. CoTS is known for its ability to survive under certain unfavourable conditions such as food scarcity. For instance, juvenile CoTS can delay diet transition and survive on suboptimal nutrient sources while awaiting improved conditions (Deaker et al. 2020). Similarly, adult CoTS can survive for extended periods without food (Chesher 1969). The conservation of relatively low methylation levels across invertebrate genomes suggests a strategy prioritising gene expression flexibility over rigid regulatory mechanisms. This configuration is linked to phenotypic plasticity and environmental adaptability, as it allows more room for inducible methylation during stress (Ardura et al. 2017; Trigg et al. 2022). For example, rapid global DNA methylation changes in response to environmental stressors have been observed in the ascidian *Didemnum vexillum*, aiding its success as an invasive species (Hawes, Tremblay, et al. 2018). In CoTS, previous research demonstrated immediate changes in gene expression in response to environmental stressors (Morin et al. 2023), suggesting a potentially flexible and dynamic epigenome that may contribute to its success as a coral reef pest. The comprehensive methylation profiles generated in this study provide a foundational data set for investigating how environmental factors influence epigenetic processes, which will deepen our understanding of the ability of CoTS to adapt to different environments and establish outbreak populations.

In our exploration of DNA methylation targets within the CoTS genome, we identified a prominent pattern of gene body methylation, a feature that appears widespread among invertebrates (Sarda et al. 2012). Our results contribute to this emerging picture by showing that in CoTS, gene body methylation is notably enriched in intronic regions. While exonic methylation is predominant in vertebrates (Feng et al. 2010), enhanced intronic methylation has also been reported in other marine invertebrates, including corals and anemones (Liew et al. 2018; Ying et al. 2022), suggesting this may be a characteristic feature in certain invertebrate lineages. Intronic methylation is less understood compared to exonic methylation, but it is being increasingly recognised for its role in fine‐tuning gene expression (Blattler et al. 2014). Although intronic regions are noncoding segments that are typically spliced out during mRNA processing, their methylation status can affect the binding of regulatory proteins and the accessibility of splicing machinery, thereby modulating mRNA isoform production (Back and Walther 2021; Espinas et al. 2020; Nam et al. 2020). By documenting this feature in CoTS, our study raises the possibility that intronic methylation represents an evolutionary adaptation essential for the development and survival of organisms in dynamic marine environments.

The elevated methylation observed within repetitive elements in the CoTS genome contributes new evidence to an evolving understanding of invertebrate epigenetics. While earlier studies suggested that transposons in invertebrate genomes were generally not specifically targeted by DNA methylation (Regev et al. 1998), more recent findings have challenged this view. For example, Ying et al. (2022) demonstrated that in cnidarians, DNA methylation predominantly targets transposons, particularly the younger and potentially more active elements. Similarly, Wang et al. (2014) documented that in the Pacific oyster *Crassostrea gigas* , DNA methylation is directed towards young repetitive elements. Our finding from CoTS not only aligns with these more recent observations but extends them to echinoderms, filling a phylogenetic gap in invertebrate methylation studies. By documenting elevated methylation in repetitive elements, this study reinforces the hypothesis that DNA methylation serves a conserved genome defence function across lineages. This protective mechanism is essential for suppressing the expression and transposition of transposable elements, thereby preserving genome stability and integrity (Bestor 2007) and preventing harmful consequences such as gene disruption, mutations and chromosomal rearrangement (Fedoroff 2012). The consistent targeting of transposons and repetitive elements across various lineages underscores a fundamental role of DNA methylation in genomic defence, reflecting a common evolutionary solution to the shared challenges posed by transposable elements.

While significant progress has been made in characterising the CoTS epigenome in the current study, certain limitations must be acknowledged. The use of related, captive‐reared individuals may constrain the generalisability of our findings, particularly for population‐level variation at loci influenced by genetic background or environmental conditions. As such, our results should be viewed as a resource‐building effort, with the controlled design enabling the development of protocols and a reference methylome to support future studies incorporating greater biological and environmental diversity. Moreover, future research should focus on how methylation patterns influence gene expression dynamics under varying environmental stressors and contribute to the species' resilience. For instance, research on the highly invasive tunicate *Ciona robusta* has demonstrated that DNA methylation aids in retaining stress adaptations and enhancing performance under fluctuating conditions (Fu et al. 2021). Conducting similar research on CoTS may lead to the development of more effective intervention strategies by better understanding the species' epigenetic stress response mechanism. Additionally, developing epigenetic biomarkers for monitoring CoTS populations could be highly beneficial. For example, epigenetic clocks, which estimate the age of organisms based on DNA methylation levels at age‐related CpG sites, have shown promise in fisheries management by providing a high‐resolution and efficient way to assess age structure (Piferrer and Anastasiadi 2023). This is particularly relevant for CoTS management, as the size‐age relationship in CoTS is highly plastic (MacNeil et al. 2017), and no validated method currently exists to accurately age wild specimens. This limitation hinders our understanding of population age structure and outbreak origins. Overall, further investigation into the epigenetics of CoTS could provide a deeper understanding of the biological adaptability driving population outbreaks and lead to the development of effective tools for population monitoring and management.

## 5 Conclusion

Our study demonstrates the robustness of ONT for comprehensive genomic and methylation analysis in nonmodel species, using CoTS as a case study. By optimising DNA extraction and size selection protocols, we achieved long reads averaging over 2 kbp, establishing a valuable framework for future long‐read sequencing of species that face challenges with high molecular weight DNA extractions. Our data cover over 90% of CpG dinucleotides in the CoTS reference genome, providing the first genome‐wide perspective of the CoTS methylome. The methylation landscape of CoTS provides empirical evidence for an intermediate position of deuterostome invertebrates in the evolutionary spectrum of DNA methylation, balancing gene expression plasticity with regulatory robustness. The CoTS genome is moderately methylated, primarily targeting gene bodies and repetitive elements, suggesting conserved mechanisms in genome regulation and defence. Additionally, we observed unique features such as intronic methylation, which may have important implications for the biological adaptability of CoTS. Overall, our comprehensive methylation profiles not only establish a foundational understanding of the CoTS epigenetic landscape but also offer a critical dataset and related protocols for future functional epigenome and comparative analyses, contributing to our understanding of DNA methylation in natural populations.

## Author Contributions

S.L.T.K., A.M.B. and S.U. conceived and designed the research. S.L.T.K. collected the samples and performed the laboratory work with support from J.Y.‐H.H. S.L.T.K. conducted the bioinformatic analyses, with input from A.M.B., C.V.R. and S.U. on results interpretation. S.L.T.K. wrote the manuscript, and all authors reviewed and approved the final version.

## Conflicts of Interest

The authors declare no conflicts of interest.

## Supporting information


**Data S1:** men70026‐sup‐0001‐Supinfo.pdf.

## Data Availability

The raw sequencing data, along with associated sample and sequencing metadata, have been deposited in the NCBI Sequence Read Archive (SRA) under BioProject accession number PRJNA1251702. Methylome data for all samples are available on Figshare (http://doi.org/10.6084/m9.figshare.27068959). The workflow guide and bioinformatics scripts are accessible on GitHub (https://github.com/sarahkwong/cots_methylome/).
