# Supplementary material for: Methylome Profiling of a Deuterostome Invertebrate Using Oxford Nanopore Technology (ONT)
Source: Mol Ecol Resour. 2025 Aug 5;25(8):e70026. doi: 10.1111/1755-0998.70026 (PMC12550476; doi:10.1111/1755-0998.70026)
Supplement: Supplementary file 1 — Data S1: men70026‐sup‐0001‐Supinfo.pdf. [file MEN-25-e70026-s001.pdf]

Supplementary Information for:

Methylome profiling of a deuterostome invertebrate using Oxford Nanopore Technology (ONT)

***Sarah Lok Ting Kwong [a, b, c]** **(ORCID 0000-0002-0933-9313)**

Alyssa Maree Budd [b, c, d] **(ORCID 0000-0002-2372-7603)**

Julia Yun-Hsuan Hung [b, c] **(ORCID 0009-0000-6809-1859)**

Cecilia Villacorta-Rath [e] **(ORCID 0000-0002-1060-5447)**

Sven Uthicke [a] **(ORCID 0000-0002-3476-6595)**

a. Australian Institute of Marine Science, PMB 3 Townsville MC, Townsville, QLD 4810, Australia
b. College of Science and Engineering, James Cook University Bebegu Yumba Campus, Townsville, QLD 4811, Australia
c. Centre for Tropical Bioinformatics and Molecular Biology, James Cook University Bebegu Yumba Campus, Townsville, QLD 4811, Australia
d. Environomics Future Science Platform, Indian Ocean Marine Research Centre, Commonwealth Scientific and Industrial Research Organisation (CSIRO), Crawley, WA, Australia
e. Centre for Tropical Water and Aquatic Ecosystem Research (TropWATER), James Cook University Bebegu Yumba Campus, Townsville, Queensland 4811, Australia

*Corresponding author; sarah.kwong@my.jcu.edu.au

Appendix S1 – Extraction and size selection of high molecular weight genomic DNA from the Pacific crown-of-thorns seastar (*Acanthaster* cf. *solaris*)

Overview

This protocol was developed after unsuccessful attempts to obtain high molecular weight DNA using the Pacbio Nanobind Tissue Kit and Qiagen Genomic-tip 20/G. Gentle handling of DNA is crucial throughout this protocol - vortexing, vigorous agitation, tube shaking, rapid or excessive pipetting should be avoided. After extraction, high molecular weight DNA between 10 – 20 kbp should be obtained, along with some small DNA fragments (< 500 bp), which are to be removed during size selection steps.

Equipment and consumables

Reagents

- MilliQ water
- 1M Tris-HCl (pH 8.0)
- 0.5M EDTA (pH 8.0)
- 5M NaCl
- 10% SDS
- Proteinase K (20 mg/mL)
- RNaseA (10 mg/mL)
- Phenol:Choloroform:Isoamyl alcohol (25:24:1)
- Chloroform:Isoamyl alcohol (24:1)
- Freshly prepared 70% and 85% ethanol
- 100% isopropanol
- Elution buffer
- SPRIselect beads (Beckman Coulter)

Consumables

- 1.5 mL and 2 mL microcentrifuge tubes

Equipment

- P1000 pipette and tips
- P200 pipette and tips
- P20 pipette and tips

- Incubator with rocking platform for heating at 65ºC
- Centrifuge
- Vortex mixer
- Magnetic rack
- Fume hood

Procedure

Tissue lysis

1. Prepare the lysis buffer by combining the following in a 2 mL tube:
   - 243 µL MilliQ water
   - 60 µL 1M Tris-HCl (pH 8.0)
   - 180 µL 0.5 M EDTA (pH 8.0)
   - 12 µL 5M NaCl
   - 60 µL 10% SDS
   - 45 µL Proteinase K (20 mg/mL)
   - 6 µL RNaseA (10 mg/mL)
2. Preheat lysis buffer at 65 ºC.
3. Add 20 mg of snap frozen pyloric caeca tissue to the preheated lysis buffer.
4. Incubate at 65ºC overnight on a rocking platform for gentle agitation. At the start of the incubation, invert the tube every 10 minutes until all visible pieces are dissolved, which should take approximately 60 minutes.

DNA extraction

Safety consideration - All steps involving phenol/chloroform extraction must be performed in a fume hood while wearing appropriate personal protective equipment.

5. Phenol:Choloroform:Isoamyl alcohol extraction
   - Add an equal volume (600 µL) of Phenol:Choloroform:Isoamyl alcohol (25:24:1) to the sample.
   - Mix by inverting the tube gently.
   - Centrifuge at 16,000 x g for 10 minutes.
   - Recover the aqueous phase (top layer, 550 µL) into a new 2 mL tube.

6. Repeat Phenol:Choloroform:Isoamyl alcohol extraction
   - Add an equal volume (550 µL) of Phenol:Choloroform:Isoamyl alcohol (25:24:1) to the sample.
   - Mix by inverting the tube gently.
   - Centrifuge at 16,000 x g for 10 minutes.
   - Recover aqueous phase (top layer, 500 µL) into a new 2 mL tube.
7. Chloroform:Isoamyl alcohol extraction
   - Add an equal volume (500 µL) of Chloroform:Isoamyl alcohol (24:1) to the sample.
   - Mix by inverting the tube gently.
   - Centrifuge at 16,000 x g for 10 minutes.
   - Recover the aqueous phase (top layer, 450 µL) into a new 2 mL tube.
8. Add 1/10 volume (45 µL) of 3M sodium acetate (NaAc) to the tube and mix by inversion.
9. Add an equal volume (495 µL) of isopropanol to precipitate the DNA. Let the tube sit at room temperature for 3 hours to allow DNA precipitation.

DNA purification

10. Centrifuge at 20,000 x g for 30 minutes at 4ºC to pellet the DNA.
11. Carefully decant the supernatant. The DNA pellet, though barely visible, should appear as a small transparent spot attached to the wall of the tube.
12. Add 1 mL of 70% ethanol to the pellet and gently flick the tube to mix.
13. Centrifuge at 18,000 x g for 10 minutes at 4ºC and carefully decant the supernatant.
14. Repeat steps 12 and 13 for a second ethanol wash.
15. Let the pellet air dry until all ethanol has evaporated, which should take approximately 10 minutes.
16. Resuspend the pellet in 30 µL of elution buffer.

Size selection

17. Vortex the SPRIselect beads bottle thoroughly to ensure resuspension.
18. Add 10 µL of SPRIselect beads to 25 µL of the DNA sample to achieve a 0.4x ratio.
19. Gently flick the tube to mix the beads and sample, incubate at room temperature for 30 seconds.
20. Place the tube on a magnetic rack and allow the beads to settle at the magnet. Once the supernatant becomes clear, promptly remove and discard the supernatant.

- Note: leaving the beads in contact with the sample for too long may result in the carryover of small fragments. Remove the supernatant as soon as it becomes clear.

21. With the tube still on the magnetic rack, add 180 µL of 85% ethanol to wash the beads. Incubate at room temperature for 30 seconds, then remove and discard the ethanol supernatant.
22. Remove the tube from the magnetic rack and add 20 µL of elution buffer to the beads. Flick the tube gently to resuspend the beads.
23. Incubate the tube at 40ºC for 10 minutes to elute the high molecular weight DNA.
24. Place the tube on a magnetic rack and allow the beads to settle at the magnet.
25. Transfer the clear eluate to a new tube for storage until ready for library preparation and sequencing.

Disclaimer

The content provided in this protocol is for informational purposes only and does not constitute legal, medical, clinical, or safety advice. This protocol is not a substitute for independent professional judgment or advice. Any actions you take or retrain from taking based on the information in this protocol are solely at your own risk. Neither the authors, contributors, administrators, nor anyone else associated with this protocol or related services can be held liable for your use or reliance on the information contained herein.

Table S1 Top 50 most highly methylated genes based on the average methylation levels from three Pacific crown-of-thorns seastar (*Acanthaster* cf. *solaris*) specimens.

**Gene ID**	**Average methylation**	**Gene description**	**Gene function**
LOC110990764	100.00	protein piccolo-like	Synaptic scaffolding, vesicle trafficking
LOC110990998	100.00	uncharacterized LOC110990998	Unknown
LOC110991011	100.00	dynein beta chain, ciliary-like	Motor protein, ciliary movement and intracellular transport
LOC110974395	98.55	5-hydroxytryptamine receptor 2-like	Neurotransmitter receptor, regulates feeding and behaviour
LOC110982886	98.33	homeobox protein MOX-2-like	Transcription factor, early embryonic development
LOC110974394	97.71	trace amine-associated receptor 13c-like	Chemoreceptor, detects environmental cues
LOC110991005	97.06	uncharacterized LOC110991005	Unknown
LOC110980001	96.93	piggyBac transposable element-derived protein 4-like	Genomic rearrangement, stress response
LOC110980949	96.86	glycine-rich RNA-binding protein-like	RNA binding
LOC110974553	96.80	guanosine-3',5'-bis(diphosphate) 3'-pyrophosphohydrolase MESH1-like	Nucleotide metabolism
LOC110990635	96.75	protein NYNRIN-like	Cellular differentiation and migration
LOC110979712	96.74	uncharacterized LOC110979712	Unknown
LOC110991013	96.67	uncharacterized LOC110991013	Unknown
LOC110975871	96.66	uncharacterized protein YwjB-like	Unknown

LOC110982312	96.62	uncharacterized LOC110982312	Unknown
LOC110981599	96.55	uncharacterized LOC110981599	Unknown
LOC110988988	96.49	uncharacterized LOC110988988	Unknown
LOC110987816	96.37	uncharacterized LOC110987816	Unknown
LOC110980655	96.37	uncharacterized LOC110980655	Unknown
LOC110976337	96.31	uncharacterized LOC110976337	Unknown
LOC110987372	96.28	uncharacterized LOC110987372	Unknown
LOC110990873	96.14	uncharacterized LOC110990873	Unknown
LOC110987426	95.92	collagen alpha-1(I) chain-like	Structural protein, extracellular matrix
LOC110975748	95.89	torsin-1A-like	Protein folding, cellular stress response
LOC110982974	95.88	piggyBac transposable element-derived protein 4-like	Genomic rearrangement, stress response
LOC110988732	95.87	mitogen-activated protein kinase 4-like	Signal transduction, cellular growth regulation
LOC110988853	95.82	uncharacterized LOC110988853	Unknown
LOC110982887	95.81	plant intracellular Ras-group-related LRR protein 5-like	Innate immunity, pathogen recognition
LOC110981280	95.72	uncharacterized LOC110981280	Unknown
LOC110980168	95.72	uncharacterized LOC110980168	Unknown
LOC110985186	95.71	uncharacterized LOC110985186	Unknown
LOC110990626	95.71	30S ribosomal protein S6-like	Ribosomal protein, protein synthesis
LOC110975872	95.65	putative protein TPRXL	Unknown

LOC110973724	95.65	hydrocephalus-inducing protein homolog, transcript variant X1	Cell adhesion, sperm-egg recognition
LOC110989512	95.60	uncharacterized LOC110989512	Unknown
LOC110984313	95.59	zonadhesin-like	Cell adhesion, reproductive function
LOC110988728	95.56	proprotein convertase subtilisin/kexin type 5-like	Protein processing, precursor cleavage
LOC110988720	95.55	uncharacterized LOC110988720, transcript variant X1	Unknown
LOC110974532	95.48	beta-3 adrenergic receptor-like	G-protein coupled receptor, signal transduction
LOC110983739	95.48	caltractin	Calcium-binding, cytoskeletal organization
LOC110986753	95.47	probable E3 ubiquitin-protein ligase HECTD4, transcript variant X2	Protein degradation, ubiquitin ligase
LOC110974243	95.46	ficolin-1-like	Innate immunity, pattern recognition
LOC110986616	95.42	uncharacterized LOC110986616	Unknown
LOC110989704	95.41	uncharacterized LOC110989704, transcript variant X1	Unknown
LOC110977620	95.41	uncharacterized LOC110977620	Unknown
LOC110984535	95.41	zinc finger protein 782-like	DNA binding, transcriptional regulation
LOC110982598	95.38	serine/threonine-protein kinase pakG-like	Cell signaling
LOC110977407	95.37	uncharacterized LOC110977407	Unknown
LOC110983035	95.37	uncharacterized LOC110983035	Unknown
LOC110989471	95.32	uncharacterized LOC110989471	Unknown
